# Effect of Resveratrol on In Vitro and In Vivo Models of Diabetic Retinophathy: A Systematic Review

**DOI:** 10.3390/ijms20143503

**Published:** 2019-07-17

**Authors:** Mario D. Toro, Katarzyna Nowomiejska, Teresio Avitabile, Robert Rejdak, Sarah Tripodi, Alessandro Porta, Michele Reibaldi, Michele Figus, Chiara Posarelli, Michal Fiedorowicz

**Affiliations:** 1 Department of General Ophthalmology, Medical University of Lublin, 20079 Lublin, Poland; 2 Institute for Ophthalmic Research, University Eye Hospital, 72076 Tuebingen, Germany; 3 Eye Clinic, University of Catania, 95123 Catania, Italy; 4 Mossakowski Medical Research Centre, Polish Academy of Sciences, 02106 Warsaw, Poland; 5 Department of Ophthalmology, Hospital C. Cantù, 20081 Abbiategrasso, Italy; 6 Department of Surgical, Medical, Molecular Pathology and of Critical Area, University of Pisa, 56126 Pisa, Italy

**Keywords:** 3,4’,5-trihydroxystilbene, eye, diabetic retinopathy, neovascularization, polyphenol, resveratrol, retina

## Abstract

A large number of preclinical studies suggest the involvement of resveratrol in the prevention and treatment of eye diseases induced by oxidative stress and inflammation. We tested the hypothesis that resveratrol influences many pathways of in vitro and in vivo models of diabetic retinopathy through a systematic literature review of original articles. The review was conducted in accordance with the PRISMA guidelines. A literature search of all original articles published until April 2019 was performed. The terms “resveratrol” in combination with “retina”, “retinal pathology”, “diabetic retinopathy” and “eye” were searched. Possible biases were identified with the adopted SYRCLE’s tool. Eighteen articles met inclusion/exclusion criteria for full-text review. Eleven of them included in vitro experiments, 11 studies reported in vivo data and 3 studies described both in vitro and in vivo experiments. Most of the in vivo studies did not include data that would allow exclusion of bias risks, according to SYRCLE’s risk of bias tool. Both in vitro and in vivo data suggest anti-apoptotic, anti-inflammatory and anti-oxidative actions of resveratrol in models of diabetic retinopathy. However, results on its anti-angiogenic effects are contradictory and need more rigorous studies.

## 1. Introduction

Resveratrol, known also as 3,4’,5-trihydroxystilbene, is a natural polyphenol that is present in various fruits and vegetable and mainly found in grapes, leading to its high concentration in wines [1]. Peanuts, blueberries, bilberries and cranberries are other available sources [2,3]. 

The concentrations of resveratrol in red wine change from 8–25 µM, depending on the types of grapes used and their culture conditions [3]. The seeds and skin of grapes are rich in resveratrol, but it has relatively poor solubility in water. On the contrary, resveratrol is soluble in low percentages of alcohol, and so it is effectively absorbed by the body from wine [4].

Resveratrol is made up of two aromatic rings connected by a methylene bridge [5]. There are two different structures of resveratrol: cis- and trans-resveratrol [5]. The cis-isomeric form had not been located in grapes, but it has been known that trans-resveratrol can become cis after isomerization when exposed to ultraviolet irradiation [6]. Jeandet et al. showed that wine may hold high amounts of the cis-isomer if it was produced in the dark without the presence of light [7]. 

The literature has reported beneficial effects of resveratrol on the human body [1]. It exhibits a wide range of biological activities, including neuro- and cardioprotection, anti-aging, anti-cancer and anti-diabetic effects [8]. Resveratrol is thought to act mainly through modulation of oxidative stress, but also by modulating other physiological processes like inflammation, cell proliferation and apoptosis or angiogenesis [8]. Moreover, several studies have highlighted the effects of resveratrol within the eye as an anti-oxidant, anti-apoptotic, anti-tumor, anti-inflammatory, antiangiogenic and vasorelaxant molecule. 

A recent review by Singh et al. [9] on clinical trials including resveratrol identified 244 finished registered clinical trials, and 27 ongoing clinical trials. The published results suggest that resveratrol improved some clinical outcomes in patients suffering from numerous diseases, including obesity, malignancies (colorectal cancer and breast cancer), hypertension, Alzheimer’s disease, stroke, cardiovascular diseases and diabetes. Moreover, the clinical data suggest that resveratrol is safe even at high doses: administration of micronized resveratrol (SRT501) in cancer patients led to no serious side effects [10]. 

There is evidence that oxidative stress and inflammation promote the initiation and progression of age-related ocular diseases, including diabetic retinopathy (DR), which leads to progressive blindness if untreated [11,12,13]. Regarding these considerations, it has been proposed that anti-oxidative and anti-inflammatory phytochemicals may have a therapeutic potential in age-related ocular disorders [14].

Therefore, among phytochemicals, it has been shown that resveratrol could prevent the progression of degenerative ocular diseases, responsible for severe sight loss [15]. 

DR is the most common cause of vision loss among working-age people, and it is defined as a diabetes-induced microangiopathy affecting the retinal vasculature [15]. This is due to poor metabolic control of circulating high blood glucose levels that leads to increased inflammatory response, ischemia and cell degeneration resulting in malfunction of the blood–retinal barrier and loss of vision [16]. 

Resveratrol seems to control glucose metabolism, decrease insulin resistance and help to prevent heart disorders, lipoprotein oxidation, apoptosis and inhibition of platelet aggregation [17]; it was suggested to be an important factor in DR. Moreover, resveratrol has been proposed in the prevention and treatment of diabetic complications [18]. However, to our knowledge, resveratrol potential in DR was not evaluated in clinical trials.

Based on these premises, it is possible to hypothesize that some of the main biological activities of resveratrol, its effects and plausible mechanisms of action as demonstrated in different in vitro and in vivo conditions could influence the eye and play a role in pathologies such as DR. 

The aim of the current systematic review was to summarize the available data in the literature about the effect of resveratrol on in vitro and in vivo models of DR. 

## 2. Results

**Figure 1:**
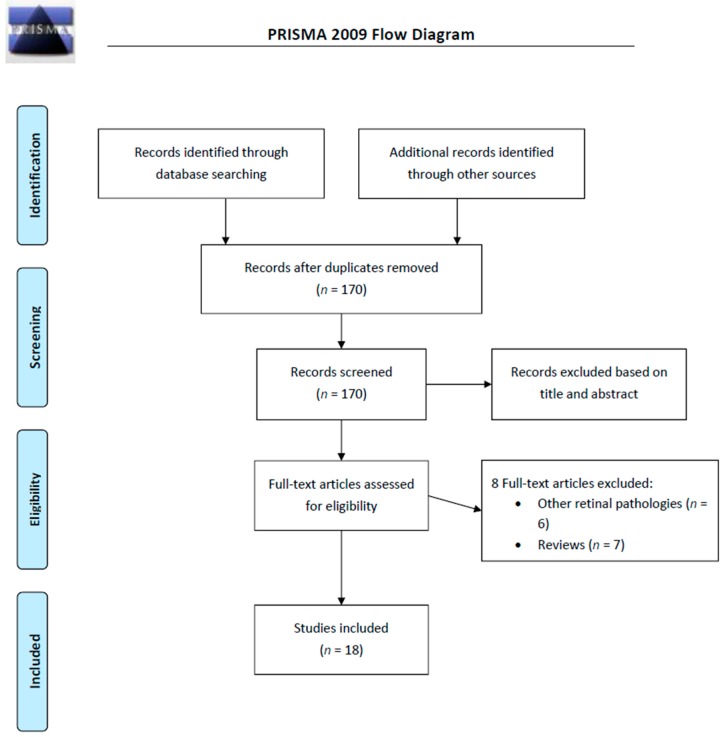
PRISMA 2009 flow diagram of the included studies.

From a total of 328 articles extracted from the initial research, there were 170 abstracts identified for screening, and 18 of these met inclusion/exclusion criteria for full-text review. We excluded 13 articles: 7 review papers and 6 because of severe bias, i.e., other ocular diseases different from DR. Our results are summarized in Figure 1. 

Eleven out of the 18 studies covered in this systematic review included in vitro experiments on human or animal cells, 11 studies included in vivo data and 3 studies described both in vitro and in vivo experiments. 

### 2.1. In Vitro Experiments

Most of the in vitro studies were performed on culture of human retinal pigment epithelial cells ARPE-19 (4 out of 11 studies), and one study was performed on human retinal endothelial cells (hRECs). One study covered non-retinal cells, i.e. peripheral blood mononuclear cells (PBMCs) isolated from proliferative DR patients [19]. All the studies using animal cells were performed on primary cultures of retinal cells: primary culture of rat Müller cells (2 studies), bovine retinal capillary endothelial cells (BRECs; 2 studies) and rat retinal endothelial cells (RREC; 1 study). Only 3 out of 11 studies provided data on identification of the cells and none of the articles using established ARPE-19 cell line provided any information on preventing misidentification.

DR was mimicked by culturing in high glucose conditions (25–33 mM glucose; 7 studies; in one of these studies additionally H_2_O_2_ exposure), exposure to PDGF-BB (20 ng/mL, 1 study) or CoCl_2_ (100–1000 μM, 1 study). In one study, there was no diabetic retinopathy-mimicking treatment [19]. The tested resveratrol concentrations were in a range from 1 µM to 10 mM. However, in only two studies from the same research group did the tested resveratrol concentrations exceed 1 mM [20,21]. In vitro studies on resveratrol effect in animal models of DR are summarized in Table 1.

#### 2.1.1. Resveratrol Effect on Cell Viability and Apoptosis

Chen et al. [22] showed that incubation of rat retinal endothelial cells (RREC) with resveratrol in normal glucose conditions did not affect cell viability up to concentration of 100 µM resveratrol. Lower resveratrol concentration suppressed high glucose-induced apoptosis (upregulation of active caspase-3). Zeng et al. [20] demonstrated that resveratrol prevented high glucose-induced retinal Müller cells apoptosis via microRNA-29b (miR-29b). Bax and specificity protein 1 (SP1) expression was downregulated by resveratrol, while Bcl-2 was upregulated. miR-29b inhibitor reversed the anti-apoptotic effect of resveratrol.

#### 2.1.2. Resveratrol Effect on ROS Production/Oxidative Stress

Chang et al. [23] demonstrated that resveratrol reduced oxidative stress in ARPE-19 cells that was induced by exposure to CoCl_2_ (hypoxic mimetic treatment). Soufi et al. [24] also showed down-regulation of high glucose-induced oxidative stress hRECs (Human Retinal Endothelial Cells) by pterostilbene, a homologous derivative of resveratrol. Li et al. [25] showed reduction of high glucose-induced intracellular ROS elevation through the activation of AMPK/Sirt1/PGC-1α pathway and apoptosis suppression in bovine retinal capillary endothelial cells (BRECs).

#### 2.1.3. Resveratrol Effect on Inflammatory Markers

One study by Losso et al. [26] on ARPE-19 cells showed inhibitory effect of resveratrol on high-glucose induced elevation of pro-inflammatory factors: decreased GJIC, secretion of cytokines IL-6 and IL-8, downregulation of Cx43, activation of TGF-β, PKCβ, and COX-2. Also, Soufi et al. [24] showed downregulation of inflammatory markers in hRECs (Human Retinal Endothelial Cells).

#### 2.1.4. Resveratrol Effect on VEGF 

A single study by Subramani et al. [27] demonstrated downregulation of VEGFR-2 and its activation, reduction of VEGF-A, and a decrease in the proliferation of cultured RPE cells.

#### 2.1.5. Other Cellular Effects of Resveratrol

Chan et al. [28] demonstrated involvement of PDGFRb, PI3K/Akt and MAPK pathways in resveratrol-driven cellular response to PDGF-BB-induced migration. Data by Chang et al. [23] CoCl_2_ (100–1000 μM) showed that resveratrol reduced hypoxia-induced secretion of HMGB1. Kowluru et al. [29] demonstrated involvement of Sirt1 activity inhibition and prevention of increase in the acetylation of p65, binding of p65 with MMP-9 promoter and activation of MMP-9. Sirt 1 involvement in resveratrol protection was also reported by Li et al. [25] and Liu et al. [19]. Zeng et al. [21] showed that resveratrol prevented high glucose-induced decrease of glutamate transporters (GLAST) expression and decrease in glutamate uptake.

### 2.2. In Vivo Experiments

The analyzed animal studies were conducted on laboratory rodents: mice (C57Bl/6—3 studies; and transgenic Nestin-Cre mice, Tie2-Cre mice—1 study) or rats (Wistar rats—4 studies; Sprague-Dawley—3 studies; Dark Agouti—1 study). Therefore, only 4 out of 11 studies were performed on pigmented strains. In 10 out of 11 studies, DR was induced by injection of streptozotocin (mostly as a single intraperitoneally or intravitreal injection in a dose ranging from 50 to 60 mg/kg body weight). In a single study [30], a model of oxygen-induced retinopathy (neonatal mice were exposed to 75% oxygen from P7 to P12) was used. Apart from one study [31], all the studies investigated changes present in the whole retina (one of these studies referred to ‘eye tissues’ [32]). Resveratrol was administered by oral route (9 studies, doses ranging from 5 to 400 mg/kg/day), intraperitoneally (1 study, 5 mg/kg/day) or by intravitreal injection (5µL of 0.1 μg/mL or 1 μg/mL solution). Bias risk in the in vivo studies is summarized in Table 2. In vivo studies on resveratrol effect in animal models of DR are summarized in Table 3.

#### 2.2.1. Resveratrol Effect on Retinal Cell Apoptosis

Four studies demonstrated that resveratrol reduced STZ-induced retinal cell apoptosis [20,22,24,33]. Chen et al. [22] demonstrated that intravitreal injection of resveratrol (5 µl of 0.1 μg/mL or 1 μg/mL solution) reduced retinal elevation of active caspase-3 after diabetes induced by streptozotocin administration. Soufi et al. in their two compatible papers [24,33] showed that prolonged oral administration of resveratrol (5/mg/kg/day for 4 months) reduced STZ-induced apoptosis (measured with cell death detection ELISA kit) and partially protected against STZ-induced retinal disorganization. Zeng et al. [20] also demonstrated that prolonged oral administration (in both tested doses of 5 and 10 mg/kg/day) of resveratrol inhibited STZ-induced apoptosis (measured with TUNEL staining) within the inner nuclear layer.

#### 2.2.2. Resveratrol Effect on Inflammatory Markers

Five of the analyzed papers reported anti-inflammatory effects of resveratrol administration. Chen et al. [22] reported that intravitreal injection of resveratrol reduced STZ-induced upregulation of several inflammatory factors (IL-1β, IL-6, TNFα, VEGF, IFN-γ and MCP-1) and reduced ox-LDL in the retina. They postulated that inflammation suppression might be driven by increased expression and activity of PON1. Kubota et al. [34] reported that resveratrol suppressed diabetes-induced upregulation of NF-κB signaling by activating the AMPK pathway. Three papers from Soufi et al. [24,33,35] showed a decrease in inflammation as a result of resveratrol administration: it prevented STZ-induced activation of NF-κB and STZ-induced upregulation of pro-inflammatory mediators (TNF-α, IL-6 and COX-2).

#### 2.2.3. Resveratrol Effect on Oxidative Stress

Two studies demonstrated attenuation of oxidative stress after resveratrol treatment in animal models of DR. Chen et al. [22] demonstrated reduction in ox-LDL level. Soufi et al. [24] showed normalization of lipid peroxidation index and oxidized to reduced glutathione ratio. They also showed increase in retinal superoxide dismutase activity after resveratrol administration.

#### 2.2.4. Resveratrol Effect on ERG Parameters

A single report provided functional evaluation of retinal function after treatment with resveratrol. Zeng et al. [21] showed that resveratrol administration provided an attenuation of diabetes-induced decreases in the amplitude of a-wave in rod response, a- and b-wave in cone and rod response or OP2 in oscillatory potentials. It also significantly repressed diabetes-induced delay in OP2 implicit times in scotopic 3.0 OPS test.

#### 2.2.5. Resveratrol Effect on Vasculature

One study by Michan et al. [30] on a model of oxygen-induced retinopathy described an increase of vaso-obliteration and no significant differences in pathologic neovascularization (although there was a trend toward suppressing). In this study, resveratrol did not show protective effects against the development of retinopathy. Kim et al. [36] demonstrated that resveratrol blocked diabetes-induced increase of VEGF expression. However, Yar et al. [32] did not detect significant changes of mRNA levels of VEGF, MMP-9, and ACE genes associated with vascular remodeling after resveratrol treatment.

#### 2.2.6. Other Effects or Mechanisms

Al-Hussaini et al. [31] reported normalization of diabetes-induced decreases in expressions of Lpl, Rdh12, Aldh1a3, Cralbp1, Cralbp2 but not Lpl, Lrat, Rdh5, Rdh10, RPE65, Rlbp1, and Rbp1 genes. Long-term (30 days) but not short term (14 days) supplementation upregulated transcription of key retinoic acid metabolism pathway enzymes. Kubota et al. [34] showed recovery of SIRT1 activity and Zeng et al. [21] reported resveratrol-induced upregulation of glutamate transporters (GLAST) and glutamine synthetase (GS) in the retina.

## 3. Discussion

DR is a priority eye disease on the VISION 2020 list, for prevention and treatment. Despite laser photocoagulation, intravitreal drugs and vitreoretinal surgery are the standard treatment care, the vision-threatening condition remains a continuous clinical challenge. Resveratrol has many mechanisms of action and it could have a role in the treatment and prevention of DR [37]. In particular, results of clinical trials on patients with type II diabetes suggest that resveratrol reduces insulin resistance [38,39,40,41], reduces oxidative stress [39] and concentration of pro-inflammatory cytokines [42].

To our knowledge, this is the first review about resveratrol and its possible mechanisms on DR. The aim of this study was to summarize all available literature regarding this topic. Eighteen studies were identified. 

Both in vitro [20,22] and in vivo [20,22,24,35] studies demonstrated that resveratrol may suppress insult-induced apoptosis of retinal cells. The proposed mechanisms include microRNA-29b (miR-29b). It might also be driven by attenuation of oxidative stress that was also shown both in vitro [23,24,25] and in vivo [22,24]. Retinal superoxide dismutase activity was also shown to be elevated after resveratrol administration. 

Reduction of high glucose-induced intracellular ROS elevation could be mediated through the activation of AMPK/Sirt1/PGC-1α pathway. Resveratrol is known as an activator of sirtuin1 (SIRT1), one of SIRT proteins, NAD+-dependent histone/protein deacetylases. SIRTs are linked with several signaling pathways regulating inflammation attenuation, DNA damage repair, apoptosis, redox status and others [43]. Resveratrol was proposed to exert its anti-inflammatory and antioxidative actions as well as suppression of tumorigenesis and vasodilation through activation of SIRT1 [9,44].

The analyzed reports also support the view that resveratrol may act through suppression of inflammatory mechanisms. Downregulation of pro-inflammatory mediators was shown both in vitro [24,26] and in vivo [22,24,34,35,36]. It is postulated that these effects might be mediated by increased expression and activity of PON1. Another proposed mechanism is suppression of diabetes-induced upregulation of NF-κB signaling (through AMPK pathway).

Finally, evidence of suppression of neovascularization by resveratrol is controversial. Only one study [27] demonstrated downregulation of VEGF and a decrease in the proliferation of cultured RPE cells. The in vivo data are not consistent. While one study [33] showed blockage of diabetes-induced increase of VEGF expression, the other two did not find any effects of resveratrol on molecular level [32] and on formation on new pathological vessels. 

Despite the limited number of studies, there are also some other limitations of these data. Most of the in vitro papers do not provide any data how cells/cell line misidentification was excluded; no guidelines like [45,46] on avoiding misidentification are cited. Moreover, a large number of animal studies we included were conducted on albino animals with compromised retinal pigment epithelium, and most likely developing several other ocular pathologies [47,48]. Another limitation is that resveratrol is rapidly metabolized and its biovailability is poor. Moreover, the activity of resveratrol metabolites is not well studied.

To evaluate animal studies, we used SYRCLE’s risk of bias tool, an adapted version of Cochrane risk of bias tool. Six out of 11 studies declare random assignment of animals to experimental groups but none of them provide any details of the procedure. None of the studies provide any details on blinding of the groups throughout the experiments. Only one study mentions a procedure for blinding of the outcome assessment. No study provided calculations of sample size. Therefore, none of the studies fully meets the current criteria of complete and transparent reporting of animal research [49]. 

To conclude, the results of in vitro and in vivo experiments seem to support resveratrol potential as a candidate for clinical trials in diabetic retinopathy. In particular, activation of AMPK/Sirt1/PGC-1α pathway could be an important mechanism mediating potential beneficial effects of resveratrol in DR. Promising clinical data in type II diabetes patients also encourage to undertake clinical evaluation of resveratrol potential beneficial effects in DR, even if the preclinical data specific for DR suffer from some limitations.

## 4. Materials and Methods

In this systematic review, we searched all available articles for each specific issue to explore the effects and the mechanism of action of resveratrol on in vitro and in vivo models of DR. This review was conducted and reported according to the Preferred Reporting Items for Systematic Reviews and Meta-Analyses (PRISMA) guidelines [50].

A literature search of all original articles published until April 2019 was performed in parallel by two authors (Alessandro Porta and Sarah Tripodi) using the PubMed database. The terms “resveratrol” was searched in combination with “retina”, “retinal pathology”, “diabetic retinopathy”, “eye”. The resulting reference lists were manually examined to identify any potential studies that were not captured by the electronic searches.

The final list of all electronic data captured, titles and abstracts were independently examined by two reviewers (Michele Reibaldi and Mario D. Toro) to identify relevant articles. Studies were considered for inclusion if they included retinal changes after resveratrol administration either in animal or in human eye with diabetic retinopathy. Then, the same reviewers registered and selected related studies that met the above inclusion criteria by examining the full text of articles. All study types were extracted. Reviews were excluded, but they were used to capture articles reported in bibliography. Only English articles were included.

Any disagreement was assessed by consensus and when it was not initially reached a third Reviewer (Michele Figus) was consulted. Two Reviewers (Michal Fiedorowicz and Chiara Posarelli) independently extracted data using an Excel sheet. 

For in vitro studies the following data were extracted: study title, author, year of publication, type of cells and their origin, data on cell identification, resveratrol concentration, laboratory techniques and major findings/mechanism of action. For in vivo studies: study title; author; year of publication; animal’s species, strain, sex and age; type of animal model of diabetic retinopathy; number of animals per group (and number of animals in total if clearly specified); route of administration and dosing of resveratrol, laboratory techniques, tissues of interest and major findings/mechanism of action.

Quality of in vivo studies was assessed, and possible biases were identified by adopting SYRCLE’s risk of bias tool for animal studies [51], as reported in Table 2.

## Figures and Tables

**Table 1:** In vitro studies on resveratrol effect on cultured retinal cells.

Author, Year (Country)	Cells	Origin	Identification of Cells/Authentication of Cell Line	Insult	Resveratrol Concentration (Incubation Time)	Laboratory Techniques	Major Findings
Chan Chi-Ming, 2013 (Taiwan, USA) [26]	ARPE-19	Human cell line (retinal pigment epithelial cells) obtained from Food Industry Research and Development Institute (Hsinchu, Taiwan)	No data	PDGF-BB (20 ng/mL) at 37 °C for 30 minutes	1, 3 or 10 µM	ECIS migration assay, MTT assay, dot binding assay, WB, in vitro scratch wound healing assay	Resveratrol inhibited PDGF- BB-induced migration and signaling in ARPE19 cells possibly through via PDGFRb, PI3K/Akt and MAPK pathways. Resveratrol had no effect on the RPE cell adhesion to fibronectin.
Chang Yo-Chen, 2017 (Taiwan) [21]	ARPE-19	Human cell line (retinal pigment epithelial cells) obtained from ATCC	No data	CoCl_2_ (100–1000 μM)—hypoxic mimetic treatment	20 µM	IP, WB, gelatin zymography, ELISA, RT PCR	Resveratrol reduced hypoxia-induced secretion of HMGB1.Oxidative and hypoxic stresses reduction; angiogenetic and fibrotic changes and tissue remodeling
Chen Yuhua, 2019 (China) [20]	Rat retinal endothelial cell (RREC) culture	Primary culture of rat cells	No data	High glucose conditions (30 mM glucose for 7 days)	10, 50, 100, 200 or 500 μM (24 h)	MTT assay, WB, RT PCR	Incubation with resveratrol did not affect cell viability up to 100 µM in normal glucose concentration conditions. Inflammation suppression and increased expression of PON1 as well as suppression of active caspase-3 upregulation driven by culturing in exposure to elevated glucose levels.
Kowluru Renu A., 2014 (USA) [27]	Bovine retinal capillary endothelial cells (BRECs)	Primary culture of bovine cells	No data	H_2_O_2_ exposure (250 µM for 1 h) and high glucose conditions (20 mM for 4 days)	25 µM	IP, RT PCR, WB, enzyme activity assay, ROS assay	Resveratrol ameliorated high glucose-induced inhibition of Sirt1 activity and prevented increase in the acetylation of p65, binding of p65 with MMP-9 promoter and activation of MMP-9.
Li Jun, 2017 (China) [23]	Bovine retinal capillary endothelial cells (BRECs)	Primary culture of bovine cells	Expression of Von Willebrand factor (IHC)	High glucose conditions (30mM glucose)	1, 5, 10 or 20μM (48 h)	Flow cytometry, RT PCR, WB	Reduction of high glucose-induced intracellular ROS elevation through the activation of AMPK/Sirt1/PGC-1α pathway and apoptosis suppression.
Liu Shulin, 2016 (China) [17]	Peripheral blood mononuclear cells (PBMCs)	19 patients with proliferative diabetic retinopathy and 20 controls	No data	No insult	10 µM (72 h)	ELISA, WB, RT PCR	IL-17 expression was upregulated and SIRT1 expression levels were decreased in the PBMCs of patients with proliferative diabetic retinopathy
Losso Jack, 2010 (USA) [24]	ARPE-19	Human cell line (retinal pigment epithelial cells) obtained from ATCC	No data	High glucose conditions (33 mM glucose)	1.25, 2.5, 5, 10 µM (9 days)	crystal violet cell viability assay, ELISA, WB, scrape-Loading/dye transfer assay	Inhibitory effect on hyperglycemia-induced inflammation in retinal pigment epithelial cells: ameliorated decreased GJIC, secretion of cytokines IL-6 and IL-8, downregulation of Cx43, activation of TGF-β,PKCβ, and COX-2.
Shen Hongjie, 2015 (China) [22]	hRECs (Human Retinal Endothelial Cells)	Human cell line (retinal pigment epithelial cells) obtained from Angio-Proteomie (USA)	No data	High glucose conditions (33 mM glucose for 72 h)	homologous derivative of resveratrol, pterostilbene 1 mM (72 hours)	MTT assay, ELISA, enzyme activity assay, ROS assay	Regulation of oxidation balance by decreasing inflammation, and further regulation retinal cells over proliferation to delay diabetic retinopathy progress.
Subramani Murali, 2017 (India) [25]	ARPE-19	Human cell line (retinal pigment epithelial cells) obtained from Karolinska Institute, Sweden	No data	Bevacizumab (0.25 mg/ml for 2 h)	100 µM (48 h)	Trypan blue assay, MTT assay, FLICA, RT PCR, BrdU assay, IHC, WB, scratch assay	Downregulation of VEGFR-2 and its activation, reduction by 50% of VEGF-A, decrease in the proliferation of cultured RPE cells, restoring the membrane integrity of blood-retinal barrier
Zeng Kaihong, 2016 (China) [19]	Rat Müller cells	Primary culture of rat cells	Müller cells were identified by expression of glutamine synthetase, vimentin and glutamate transporter (IHC)	High glucose conditions (25 mM for at least 3 days)	10, 20 or 30 mM (for at least 3 days)	Glutamate uptake assay, enzymatic activity assay, IHC, RT PCR	Resveratrol prevented high glucose –induced decrease of glutamate transporters (GLAST) expression and decrease in glutamate uptake.
Zeng Kaihong, 2017 (China) [18]	Rat Müller cells	Primary culture of rat cells	Müller cells were identified by expression of glutamine synthetase, vimentin and glutamate transporter (IHC)	High glucose conditions (25 mM for at least 3 days)	10, 20 or 30 mM (for at least 3 days)	RT PCR, enzymatic activity assay, IHC	Resveratrol prevented high glucose-induced retinal Müller cells apoptosis via microRNA-29b (miR-29b): decreased Bax and specificity protein 1 (SP1) expression and increased Bcl-2. miR-29b inhibitor reversed the anti-apoptotic effect of resveratrol.

**Table 2:** Summary of risk of bias analysis in studies on resveratrol effect in animal models of diabetic retinopathy. None of the studies provided data on allocation concealment or blinding the groups throughout the experiment.

	Al-Hussaini et al. [29]	Chen et al. [20]	Kim et al. [34]	Kubota et al. [32]	Michan et al. [28]	Soufi et al. [22]	Soufi et al. [31]	Soufi et al. [33]	Yar et al. [30]	Zeng et al. [19]	Zeng et al. [18]
**Random allocation**	No data	No data	No data	No data	No data	Yes	Yes	Yes	Yes	Yes	Yes
**Blinding of outcome assessment**	No data	No data	No data	No data	Yes	No data	No data	No data	No data	No data	No data
**Sample size calculation**	No	No	No	No	No	No	No	No	No	No	No

**Table 3:** In vivo studies on resveratrol effect in animal models of diabetic retinopathy.

Author, Year (Country)	Animals	Sample Size (n)	Animal Model	Resveratrol Dosing)	Follow Up	Laboratory Techniques	Tissues Studied	Major Findings
Al-Hussaini Heba 2018 (Kuwait) [29]	Male Dark Agouti rats (16 weeks old)	*n* = 5, *n* = 6 or *n* = 10 per group	Streptozotocin (60 mg/kg body weight; single, ip)	5 mg/kg; i.p., daily (6 days a weeks) starting from day 2 after STZ till the end of experiment	14 days or 30 days	RT PCR, microarrays, WB	RPE	Exageration of type 1 diabetes-induced gene inhibition (normalization of diabetes-induced decreases in expressions of Lpl, Rdh12, Aldh1a3, Cralbp1, Cralbp2 but not Lpl, Lrat, Rdh5, Rdh10, RPE65, Rlbp1, and Rbp1 genes). Long term (30 days) but not short term (14 days) supplementation upregulated transcription of key retinoic acid metabolism pathway enzymes
Chen Yuhua, 2019 (China) [20]	Male Sprague-Dawley rats (14 weeks old)	*n* = 5 or *n* = 4 per group	Streptozotocin (60 mg/kg body weight; single, iv)	intravitreal injection 2 wks after STZ (0.1 μg/mL or 1 μg/mL in one eye) or daily intravenous injections (5, 10 or 50 μg/kg/d) for 12 weeks.	12 weeks	RT PCR, WB, enzymatic activity assay, ELISA	Whole retina	Inhibition of apoptosis (lower expression of active capsase 3), reduction of inflammation: reduced inflammatory factors, reduced ox-LDL. Inflamation suppression might be driven by increased expression and activity of PON1.
Kim Young Hee, 2012 (Korea) [34]	Male C57BL⁄ 6 mice (8 weeks old)	*n* = 4 per group	Streptozotocin (55 mg/kg body weight; for 5 consecutive days, ip)	Oral administration one month after the last injection of STZ, 20 mg/kg once daily for 4 weeks	8 weeks	Fluorescein angiography, Evans blue BBB leakage assay, IHC, WB, ELISA	Whole retina	Blockage of diabetes-induced early vascular lesions and pericyte loss. Blockage of diabetes-induced increase of VEGF expression.
Kubota Shunsuke, 2011 (Japan) [32]	C57BL/6 mice (6 weeks old, sex not specified)	*n* = 7 or *n* = 8 per group	Streptozotocin (60 mg/kg body weight; for 5 days, ip)	Oral administration (by gastric intubation) seven weeks after the first injection of STZ, 50 mg/kg daily for 7 days		WB, ELISA, enzymatic activity assay, perfusion labeling	Whole retina	Anti-inflammatory effects, suppression of leukocyte adhesion to the retinal vasculature Normalization of diabetes-induced AMPK deactivation, recovery of SIRT1 activity. Suppression of diabetes-induced upregulation of NF- κB signaling by activating the AMPK pathway. Suppression of leukocyte adhesion to the retinal vasculature.
Michan Shaday, 2014 (USA, Mexico, Australia) [28]	Nestin-Cre mice, Tie2-Cre mice, and C57Bl/6J mice (neonatal, both sexes?)	*n* = 6 or *n* = 8–20 per group	Oxygen induced retinopathy (neonatal mice exposed to 75% oxygen from P7 to P12)	Oral administration, micronized formulation of resveratrol, SRT501 (400 mg/kg body weight) given daily from P5 to P17.	13 days	IHC, HE staining, RT PCR, WB	Whole retina	Increase of vaso-obliteration and no significant differences in pathologic neovascularization, (although there was a trend toward suppressing). Resveratrol did not show protective effects against the development of retinopathy.
Soufi Farhad Ghadiri, 2012 (Iran) [31]	Male Wistar rats (320–350g)	48	Streptozotocin (50 mg/kg body weight single, ip)	Oral administration 5/mg/kg/day	4 months	TBARS assay, ELISA	Whole retina	Anti-hyperglycemic and antioxidant effects, reduction of inflammatory mediators (TNFα, IL-6 and NF-κB). Reversion of apoptosis. Prevention from disarrangement and reduction in thickness of retinal layers.
Soufi Farhad Ghadiri, 2012 (Iran) [22]	Male Wistar rats (320–350g)	48	Streptozotocin (50 mg/kg body weight; single, ip)	Oral administration 5/mg/kg/day for 4 months	4 months	HE staining ELISA, enzymatic assay	Whole retina	Reduction of apoptosis, oxidative stress and anti-hyperglycemic effect with decrease of inflammation (prevents STZ-induced activation of NF-κB).
Soufi Farhad Ghadiri, 2015 (Iran) [33]	Male Wistar rats (12-week old, 320-350 g)	*n* =6 or *n*=12 per group	Streptozotocin (50 mg/kg body weight; single, ip)	Oral administration 5 mg/kg/day for 4 months	4 months	RT PCR, ELISA	Whole retina	Inhibition of STZ-induced enhancement of retinal apoptosis and upregulation of pro-inflammatory mediators (TNF-a, IL-6 and COX-2), reduction of STZ-induced retinal NF-κB activity and mRNA expression.
Yar Seda Atiye, 2012 (Turkey) [30]	Male Wistar rats (3-month old, 250-300g)	*n* = 24 (*n* = 6 per group)	Streptozotocin (55 mg/kg body weight; single, ip)	Oral administration 10 mg/kg/day for 4 weeks starting 4 weeks after STZ	8 weeks	RT PCR, biochemical measurements	Eye tissues	Suppression the expression of eNOS, but mRNA levels of VEGF, MMP-9, and ACE genes associated with vascular remodeling did not change significantly.
Zeng Kaihong, 2016 (China) [19]	Sprague–Dawley rats (14-week old, sex not specified)	*n* = 408 (*n* = 68 per group)	Streptozotocin (60 mg/kg body weight; single, ip)	Oral administration 5 or 10 mg/kg/day for 1, 3 5 or 7 months	1, 3 5 or 7 months	ERG, RT PCR, WT	Whole retina	Attenuation diabetes-induced decreases in amplitude of a-wave in rod response, a- and b-wave in cone and rod response or OP2 in oscillatory potentials, significantly repressed diabetes-induced delay in OP2 implicit times in scotopic 3.0 OPS test. Upregulation of glutamate transporters (GLAST) and glutamine synthetase (GS).
Zeng Kaihong, 2017 (China) [18]	Male Sprague–Dawley rats (14-week old, sex not specified)	*n* = 408 (*n* = 68 per group)	Streptozotocin (60 mg/kg body weight; single, ip)	Oral administration 5 or 10 mg/kg/day starting 3 days after STZ	1, 3 5 or 7 months	TUNEL staining, IHC, RT PCR, WB, caspase-3 assay	Whole retina	Suppression of the elevated levels of plasma glucose and fructosamine in STZ-treated rats. Suppresion of STZ-induced retinal cells apoptosis.
